# CRISPR-assisted test for *Schistosoma haematobium*

**DOI:** 10.1038/s41598-023-31238-y

**Published:** 2023-03-27

**Authors:** Dounia Cherkaoui, Silvia G. Mesquita, Da Huang, Elena B. Lugli, Bonnie L. Webster, Rachel A. McKendry

**Affiliations:** 1 London Centre for Nanotechnology, University College London, London, WC1H 0AH UK; 2 Division of Medicine, University College London, London, WC1E 6BT UK; 3 Wolfson Wellcome Biomedical Laboratories, Department of Science, Natural History Museum, Cromwell Road, London, SW7 5BD UK; 4 René Rachou Institute, Oswaldo Cruz Foundation, Belo Horizonte, Minas Gerais Brazil; 5 London Centre for Neglected Tropical Disease Research (LCNTDR), London, W21 PG UK

**Keywords:** Biosensors, Parasitic infection

## Abstract

Schistosomiasis is a major neglected tropical disease targeted for elimination as a public health issue by 2030, however there is an urgent need for more sensitive and specific diagnostic tests suitable to resource-limited settings. Here we developed CATSH, a CRISPR-assisted diagnostic test for Schistosoma haematobium, utilising recombinase polymerase amplification, Cas12a-targeted cleavage and portable real-time fluorescence detection. CATSH showed high analytical sensitivity, consistent detection of a single parasitic egg and specificity for urogenital *Schistosoma* species. Thanks to a novel CRISPR-compatible sample preparation developed using simulated urine samples containing parasitic eggs, CATSH had a sample-to-result within 2 h. The components of CATSH can be lyophilised, reducing cold chain dependence and widening access to lower and middle-income countries. This work presents a new application of CRISPR diagnostics for highly sensitive and specific detection of parasitic pathogens in remote areas and could have a significant impact on the elimination of neglected tropical diseases.

## Introduction

Schistosomiasis is a parasitic disease, also classified as a neglected tropical disease (NTD)^1^, caused by a trematode fluke from the *Schistosoma* genus. Amongst the most prevalent human parasitic diseases after malaria^2^, schistosomiasis still affects close to 240 million people worldwide, with approximately 90% living in sub-Saharan Africa, and deaths due to the disease may be as high as 200,000 every year^3^. It is common in children in low- and middle-income countries (LMICs) and is associated with poor growth and development in children^4^. This waterborne infection can lead to infection in the urinary tract or intestine, and if undetected can lead to liver damage, kidney failure, infertility and bladder cancer^1,5^. *Schistosoma haematobium* (*S. haematobium*) causes the urogenital disease^6^. In addition to chronic conditions affecting the kidneys, bladder and liver, infections with *S. haematobium* may result in genital schistosomiasis causing reproductive complications, increased risk for HIV transmission, infertility and cervical cancer^7^.

The World Health Organisation (WHO) has set a roadmap for global elimination of schistosomiasis as a public health problem by 2030^8^, however many challenges remain, in particular access to high performance point-of-care (POC) diagnostic tests.

An effective drug for schistosomiasis (praziquantel) exists and is being distributed through mass drug administration programmes in endemic regions^9^. New guidelines by the WHO recommend the use of a test-and-treat approach for endemic communities with low *Schistosoma* spp. prevalence (< 10%) instead of traditional mass drug administration^10^. More targeted use of praziquantel may enhance effective control and elimination strategy^8^.

The current reference method for schistosomiasis diagnosis is microscopic examination of urine or stool samples by a trained technician, with visual egg count to assess the intensity of infection^1,3^. However, microscopy lacks sensitivity for low intensity infections, which can be as low as 1 egg per 10 mL of urine sample^11^. Thus this method is not sufficient to provide true prevalence data of light infections, particularly where elimination is being targeted^12^. Alternative laboratory tests for schistosomiasis exist but they come with both advantages and drawbacks.

Antigen tests for the detection of active *S. haematobium* infections, targeting the circulating anodic antigen (CAA), reported high sensitivity and specificity compared to the traditional microscope method, but it is not yet a rapid diagnostic test amenable to rapid POC^13,14^. Although the POC circulating cathodic antigen (POC-CCA) test is a commercially available diagnostic, it has shown not to reliably detect *S. haematobium* infections^15,16^.

Real-time PCR is traditionally sensitive and specific and has been applied to diagnose schistosomiasis^17–19^. In addition to having superior sensitivity and specificity compared to microscopy, PCR testing has shown to be more robust in detection of day-to-day parasitaemia variation and a powerful tool for monitoring treatment response^19^. Despite their accuracy, there are critical impediments for using PCR-based assays for schistosomiasis diagnosis, such as the requirement for a thermocycler, trained technicians, access to a reliable power-supply and cold chain for the reagents.

Altogether, current diagnostic methods for *S. haematobium* either lack sensitivity and specificity (microscopy and POC-CCA test) or are not suitable for testing in low-resource settings (CAA test and real-time PCR).

Recent advancements towards simplifying nucleic acid testing platforms for POC testing offer new tools in this area. Fast isothermal assays, namely loop-mediated isothermal (LAMP)^20–22^ and recombinase polymerase amplification (RPA)^23–26^, have been applied for the detection of *Schistosoma* genomic DNA (gDNA). Isothermal amplification methods are cheaper, simpler, more suitable for diagnosis in resource-limited settings compared to PCR, and more sensitive than traditional microscopy^22,27^. Reported RPA-based assays for schistosomiasis achieved high sensitivity and may close the gap for molecular testing in remote areas, but tests targeting the Dra1^23–25^ DNA region (a highly repetitive repeat region with the *S. haematobium* genome) showed cross-reactivity between *S. haematobium* and *S. mansoni*^25,26^.

CRISPR (clustered regularly interspaced short palindromic repeats) diagnostics, a cutting-edge biosensing technology, has shown promising applications for a range of diseases, including viral^28–30^ and bacterial^30^ infections. However, there are only few applications of CRISPR tests for clinical diagnosis of parasitic diseases^31–33^, including NTDs^28,34,35^, despite the huge burden worldwide. Within the CRISPR/Cas endonucleases family, Cas12a can be programmed with a customised CRISPR RNA (crRNA) to recognize and cis-cleave a double-stranded DNA (dsDNA) sequence, which activates collateral trans-cleavage of single-stranded DNA FAM-quencher (ssDNA-FQ) reporters^36^. CRISPR-based platforms like DETECTR^36^, SHERLOCK^37^, HOLMES^38^ and CARMEN^39^, have been developed. DETECTR (DNA endonuclease-targeted CRISPR trans reporter) integrates pre-amplification of nucleic acid targets using an isothermal method, such as RPA, prior to detection of amplicons with a crRNA-guided Cas12a. Similarly to other molecular assays, there are some bottlenecks to make CRISPR a POC method, such as the temperature stability of the primary components and the requirement for complex sample preparation which could limit its use in real-world settings and LMICs^40–42^.

**Figure 1:**
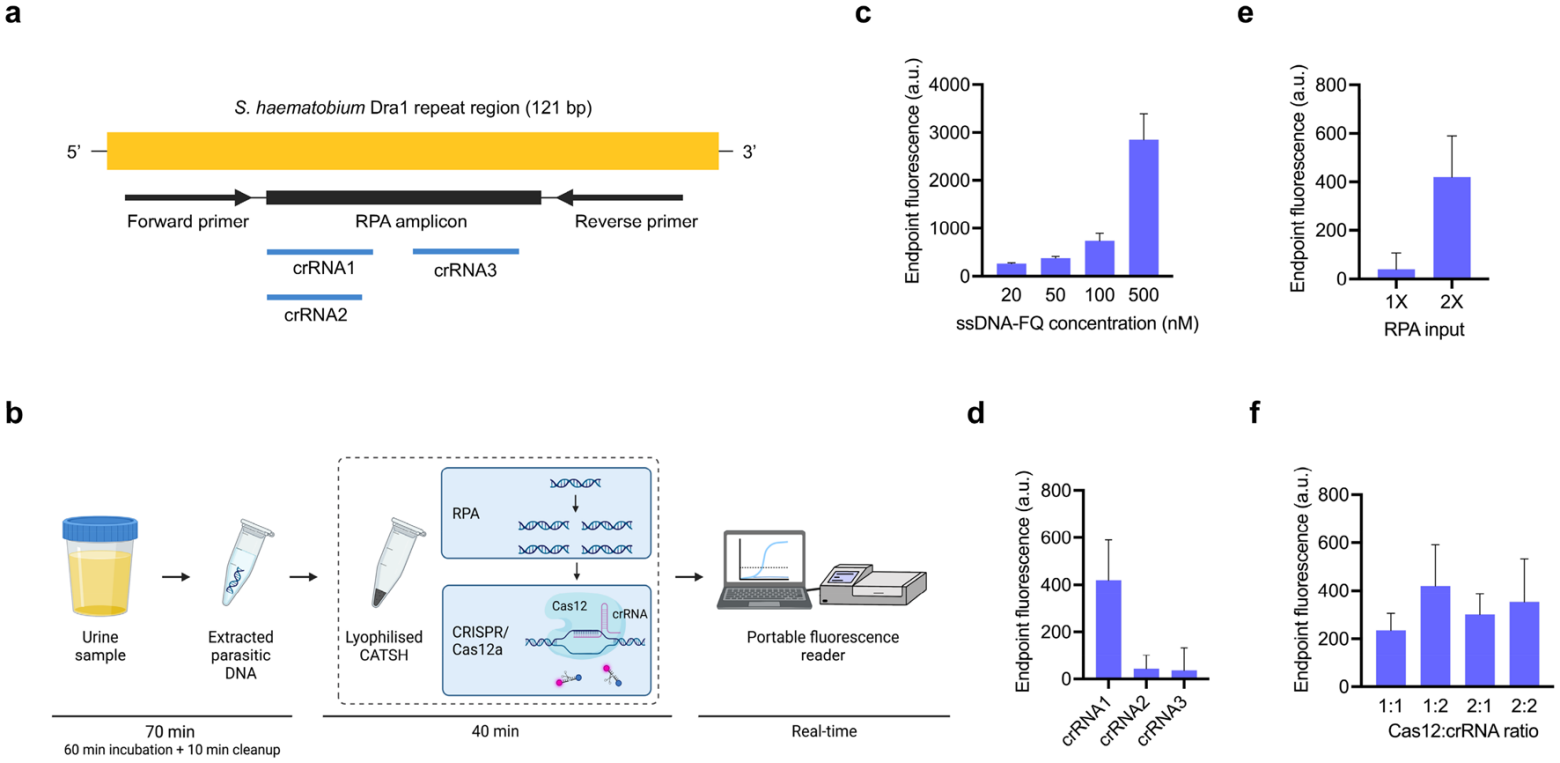
CATSH design and optimisation. (**a**) Visualisation of the targeted *S. haematobium* Dra1 repeat region with RPA primers, amplicon and crRNAs. (**b**) Schematic of the proposed CATSH workflow. Preparation of urine samples with in-house CATSH-compatible protocol, followed by CATSH testing. Real-time fluorescence is recorded on a portable reader. (**c**) Optimisation of ssDNA-FQ concentration. (**d**) Comparison of three crRNAs. (**e**) Optimisation of RPA input. (**f**) Optimisation of Cas12a to crRNA ratio. All reactions were run in five repeats. Bars represent the average endpoint fluorescence after background subtraction and error bars represent the standard deviation between repeats.

Herein, we exploited and optimised an all-in-one DETECTR protocol^43^ to develop CATSH, a CRISPR-assisted test for *S. haematobium*, for molecular diagnosis of urogenital schistosomiasis. We built on the RPA assay targeting the Dra1^23–25^ DNA region (Fig. 1a) to develop, test and optimise CATSH (Fig. 1b). Furthermore, we explored innovative sample preparation methods and reagent lyophilisation to support the use of this technology in resource-poor settings. To the best of our knowledge this is the first reported CRISPR-based clinical diagnostic for schistosomiasis.

## Results

### Optimisation of the one-pot CATSH assay

The assay was first optimised to comprise all reagents in a one-pot format. First, we optimised the concentration of ssDNA-FQ (Component B) which had a direct impact on the intensity of the fluorescence signal (Fig. 1c). The fluorescence signal increased with ssDNA-FQ concentration, with the highest signal achieved using a stock concentration at 500 nM. However, because the fluorescence reader saturates just below 5000 (A.U.), we decided against using a too high concentration of ssDNA-FQ to avoid saturating the signal when testing highly concentrated samples. Reader saturation would flaw the use of endpoint fluorescence as it would cap it to an arbitrary value and impede any possible quantitative or semi-quantitative analysis in the future. Therefore, 100 nM ssDNA-FQ was chosen as the optimal concentration.

Of the three crRNAs tested (Fig. 1d), crRNA1 was selected for the final CATSH assay as it produced a significantly higher (one-way ANOVA, *p*-value < 0.05) average endpoint fluorescence compared to crRNA2 and crRNA3. Doubling the RPA input (14 µL of Component A) was necessary to trigger efficient target pre-amplification, while using a 1× RPA input (7 µL of Component A) showed low amplification efficiency, resulting in a low fluorescence signal (Fig. 1e).

Finally, the Cas12a to crRNA ratios tested did not produce significantly different (one-way ANOVA, *p*-value = 0.2) average endpoint fluorescence (Fig. 1f). The 1:2 Cas12a to crRNA ratio (50 nM Cas12a for 100 nM crRNA) produced the highest endpoint fluorescence, thus it was chosen for the optimised assay. Addition of 1 µL of magnesium acetate (MgOAc) was sufficient to efficiently start the CATSH reaction.

### Analytical sensitivity and detection of *S. haematobium* eggs

**Figure 2:**
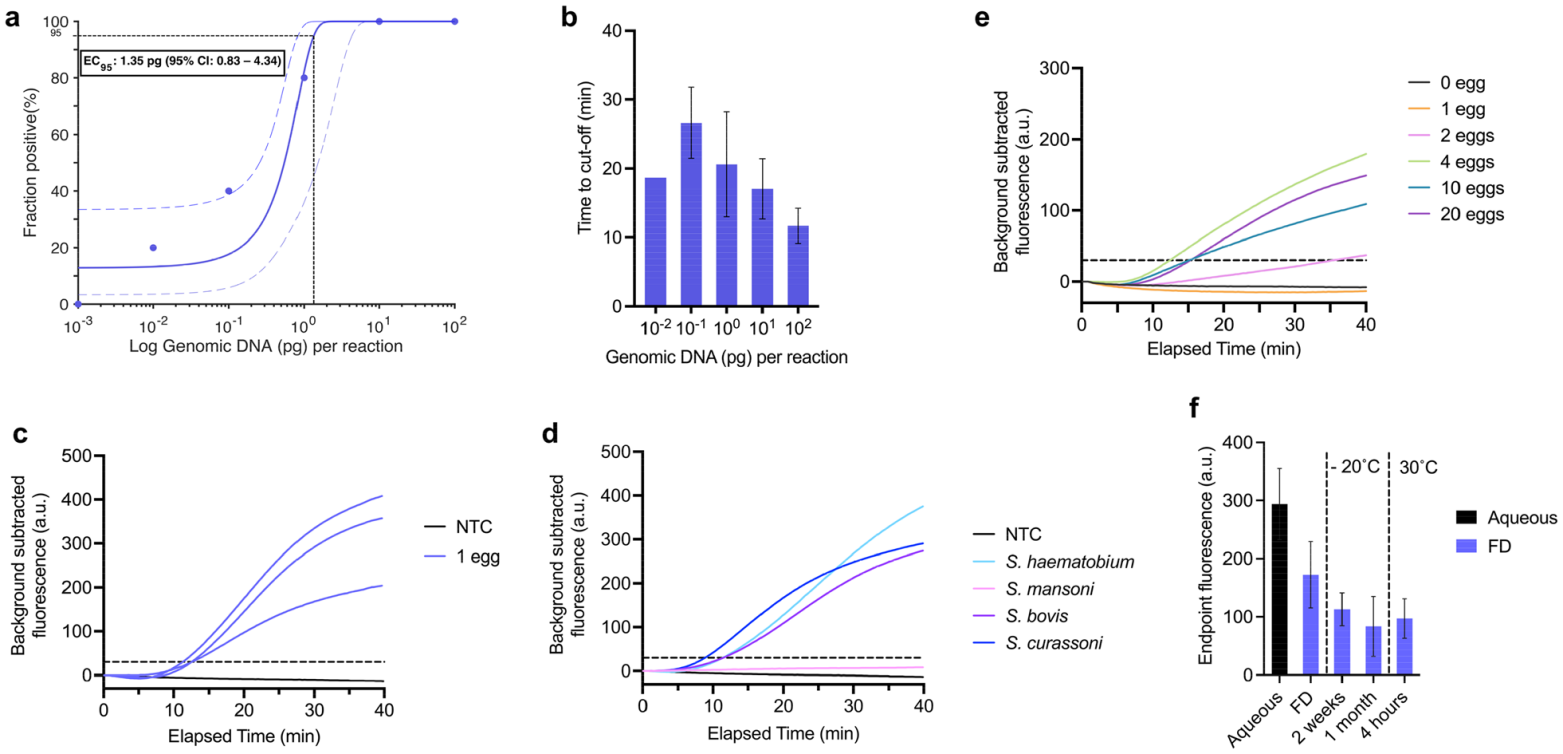
Evaluation of CATSH. (**a**) Calculation of analytical sensitivity (EC_95_) with genomic DNA using the fraction positive (dots) calculated with five replicates for each concentration tested. The Probit regression was plotted with the 95% confidence interval (dashed lines). (**b**) Time to cut-off for positive reactions. Bars represent the average time to cut-off and error bars represent the standard deviation between positive reactions. (**c**) Detection of a single parasitic egg in spiked buffer. Three individual repeats are plotted and dashed line show the fluorescent cut-off (= 30). (**d**) Species specificity tested with *S. haematobium*, *S. mansoni*, *S. bovis* and *S. curassoni*. Individual reactions are plotted. (**e**) Detection of parasitic eggs after in-house extraction from simulated urine sample. Individual reactions are plotted. (**f**) Thermostability of freeze-dried CATSH reactions. For each condition, three repeats were carried out. Bars represent the average endpoint fluorescence and error bars represent the standard deviation between repeats.

The analytical sensitivity of the CATSH assay was defined as the EC_95_, which is the lowest concentration of *S. haematobium* gDNA per reaction that can be detected ≥ 95% of the time. The fluorescence cut-off calculated from non-template control (NTC) reactions was equal to 30. The analytical sensitivity of CATSH was equal to 1.35 pg (95% CI: 0.83–4.34) of *S. haematobium* gDNA per reaction (Fig. 2a). The smallest amount of gDNA detected was 10 fg per reaction with a 20% fraction positive (1 in 5 repeats). We observed a decreasing trend of the time to cut-off (the time needed for the signal to cross the cut-off) inversely proportional to the amount of gDNA, with the exception of the time to cut-off when using 10 fg which differed from this trend (Fig. 2b).

The assay rapidly and consistently detected a single *S. haematobium* egg, added directly to the reaction, in an average time of 12 min (Fig. 2c).

### Species specificity

The cross-reactivity experiments showed that CATSH was able to detect *S. haematobium*, *S. bovis* and *S. curassoni* (Fig. 2d). No detection was observed with *S. mansoni*, an endemic *Schistosoma* species causing intestinal schistosomiasis.

### In-house CATSH sample preparation with simulated urine samples

Crude simulated urine sample containing no egg or one egg were tested by CATSH. We observed that crude urine disrupted CATSH components and detection of one egg was not possible with unprocessed urine (Supplementary Fig. 1). Two commercial nucleic acid extraction kits were tried but did not produce good results in our hands, showing incompatibility with CATSH as no signal above fluorescence threshold was detected even with highly concentrated simulated samples (Supplementary Fig. 1). In the literature, we did not find CRISPR-compatible sample preparation methods for parasitic detection in urine samples.

To overcome this challenge, an in-house method was developed to prepare simulated urine samples prior to CATSH. We observed that the sample incubation time with molecular transport medium (MTM) seemed to improve the detection with CATSH (Supplementary Fig. 2). Indeed, the sample preparation with a 1 h incubation resulted in successful detection of simulated samples containing 2, 4, 10 and 20 eggs, whereas samples prepared with a short 5-min incubation were not detected by CATSH. Thus, a longer incubation of 1 h was selected, followed by a 10-min centrifugal clean-up and concentration, still keeping the sample-to-result under 2 h. The final CATSH platform with a 70-min sample preparation method was able to detect as little as 2 *S. haematobium* eggs in a simulated urine sample using the previously set fluorescence cut-off (Fig. 2e).

### Lyophilisation of CATSH

The freeze-dried reactions showed a recovery of approximately 60% of the pre-lyophilisation endpoint fluorescence and with comparable time to cut-off (Fig. 2f). The properly packaged freeze-dried reactions (Supplementary Fig. 3) were still able to detect 10 pg gDNA after being stored in the freezer (−20 °C) for at least one month, however a decreased endpoint fluorescence was observed over time. The lyophilised CATSH reactions retained satisfactory activity and were able to detect 10 pg gDNA after being left 4 h at 30 °C.

## Discussion

We report the development of CATSH, an optimised one-tube CRISPR-based diagnostic tool for urogenital schistosomiasis, marking an important milestone for CRISPR-diagnostics for NTDs. The CRISPR/Cas12a component of CATSH detected target amplicons, followed by indiscriminate collateral cleavage of ssDNA-FQ reporters. This cleavage produced a fluorescent signal confirming the presence of parasitic DNA. CATSH consistently detected low levels of gDNA and a single *S. haematobium* egg in buffer. The assay was specific to *S. haematobium* group species and did not cross-react with the co-endemic intestinal species, *S. mansoni*. A novel sample preparation was developed, allowing CATSH to detect as low as 2 eggs in a simulated urine sample.

The lyophilisation of the CATSH reagents maintained a satisfactory fluorescent activity after long-term (1 month) storage in the freezer and short-term exposure to high temperatures (30 °C).

The analytical performance of CATSH showed that its limit of detection was a single unprocessed egg or 2 eggs that had been spiked in donor urine, which was then subsequently prepared using an adequate methodology. *S. haematobium* infections with 1–49 eggs/10 mL of urine are classed by WHO as low intensity^10^, and so our limit of detection of 1 or 2 eggs falls within this range. However, it should be acknowledged that when working with large volumes of clinical urine samples (e.g., 10 mL) a pre-concentration step (e.g., centrifugal concentration, gravity sedimentation or urinary egg filtration^44^) would need to be added to the CATSH workflow to capture the 1 or 2 eggs present within the sample. Using the current traditional diagnostic microscopy method, detection of a couple of parasitic eggs in a urine sample is challenging and egg count reported with this method may not reflect true prevalence.

CATSH analytical performances were compared to other available molecular methods. A fluorescence RPA-based assay for *S. haematobium* reported a lower detection limit of 1 fg gDNA in 10 minutes^23^. Another rapid RPA assay with detection by lateral flow showed it could detect 100 fg of *S. haematobium* gDNA in 10 min^25^. RPA assays previously reported achieved similar or superior detection limits, and faster results compared to CATSH. However, consistent detection with replicates at this low level of concentration was not previously demonstrated for RPA tests^23,25^; herein the analytical sensitivity of CATSH was reported using a statistical method and demonstrated consistent detection (< 5% false negative) of low picogram gDNA in 40 min and a single egg in 12 min average time. The lower limit of detection of CATSH was the same as a reported real-time PCR assay also targeting Dra1, equal to 10 fg^45^.

Due to its ability to detect other *S. haematobium* group species, namely *S. bovis* and *S. curassoni* (livestock species), this assay has the potential to be integrated into One Health schistosomiasis strategies^46,47^. Additionally, this uniformity across the *S. haematobium* group species means that introgressed forms also found in human infections will also be detected. Diagnosis to inform on treatment (which is independent of species or disease type) needs a diagnostic that is genus specific and so cross-reactivity with other closely related *Schistosoma* species is not a limiting factor. Moreover, we confirmed that CATSH does not cross-react with the endemic human intestinal species, *S. mansoni*; hence the test provides a specific diagnostic for urogenital schistosomiasis.

The lateral flow-based RPA assay reported by Rosser et al. had a low-cost, user-friendly and extremely portable detection method using a lateral flow, though it showed cross-reactivity with *S. mansoni*^25^ not providing specific detection of *S. haematobium* group species. Moreover, thanks to the one-pot design and in-tube fluorescence reading, there is no tube re-opening with CATSH. This considerably limits the risk of contamination, more probable with a lateral flow test readout where tubes must be re-opened post-amplification.

When testing simulated urine samples with CATSH, we observed that urine components affected the CRISPR complex, and several protocols were tried to deactivate these inhibitors. MTM has been effectively used for inactivation, stabilisation and transport of samples for instance during the COVID-19 pandemic^48^ and for some bacterial infections^49^. An in-house extraction protocol using MTM for the preparation of urine samples was successfully developed. This novel CRISPR-compatible method offers an additional sample preparation method to the CRISPR toolkit for nucleic acid extraction and detection in complex physiological matrices such as urine. We note the limitations of our current sample clean-up method which uses MTM, a purification column (DNeasy) and requires a centrifuge, an instrument which is not compatible for field testing. In future, further development of the method is needed for example to overcome the need for a centrifuge for sample preparation by investigating portable bead-based purification methodologies, resulting in a more feasible POC assay.

The diagnostic developed herein meets the WHO target product profile^50^. This target product profile describes a test that should be low-cost, easy-to-use by field workers (not requiring more than one day of training) and the readout system should be simple, portable and, if needed, battery powered. Furthermore, the amount of time to perform the assay (including sample collection, processing, assay time and result interpretation) should be less than one day so that treatment could be given to patients in one visit to (or by) the clinic. The total cost of reagents was estimated to £3 (~ 3.75 USD) per test (Supplementary Table 1). The test was made user-friendly, requiring minimal number of steps thanks to a simplified one-pot formulation with a sample-to-result within 2 h. The readout was achieved on a portable fluorescence reader with isothermal heating, able to run on an external battery for hours (~ 3 h autonomy reported for this reader but other readers have longer autonomy), hence not dependent on constant power supply.

The CATSH freeze-dried reactions could be used quickly with minimal labour by resuspension of the pellet with nuclease-free water, addition of MgOAc and sample. Altogether, the outlined features of CATSH make it more suited than PCR assays for *S. haematobium* to be deployed in resource-limited settings.

Lyophilising (freeze-drying) CATSH reagents was investigated to facilitate transportation, increase heat-stability and eventually enable field-testing. The formulation of freeze-dried pharmaceutical products, including mRNA vaccines, and diagnostics has many advantages, especially for increased portability, shelf-life, heat-stability, removing the need for ultralow temperature freezers^51^.

CATSH is one of the rare applications of CRISPR-based diagnostics for parasitic diseases, especially for NTDs. The development of a test for urogenital schistosomiasis with high performances, but adapted to low-resource settings, was investigated to highlight the potential of CRISPR for NTDs testing, as an additional tool to achieve elimination.

We showed that CRISPR-based diagnostics are amenable to detect parasitic infections, of which many are NTDs, and unlike PCR, the assay can be developed into a format suitable for POC testing in endemic countries. Furthermore, CRISPR technologies can achieve clinically relevant sensitivity (here detection of 2 eggs in urine) and a superior specificity compared to rapid isothermal methods alone, to eliminate cross-reactivity with closely related *Schistosoma* species causing intestinal schistosomiasis.

The limitations of this study include a small number of simulated urine samples and no clinical samples. Further optimisation of the lyophilised CATSH, for instance using lyoprotectants^52^, may be needed before field study. In future work, we plan to validate the lyophilised CATSH platform in a field study benchmarking the test to other diagnostics, including microscopic examination, CAA antigen testing and PCR. The clinical validation will be performed in an area with light-intensity infections, for instance in Zanzibar, to demonstrate the high sensitivity of the CRISPR-assisted assay. Ultrasensitive, low-cost, field-deployable CRISPR diagnostics for the detection of parasitic infections in resource-limited settings could become a pivotal tool to achieve the elimination of schistosomiasis and other NTDs (e.g., human African trypanosomiasis, leishmaniasis, onchocerciasis, lymphatic filariasis) as public health problems.

## Materials and methods

### Reagents

RPA TwistAmp basic kit were purchased from TwistDx (cat. No. TABAS03KIT). *Lachnospiraceae bacterium* Cas12a (Cpf1) was purchased from New England Biolabs (cat. no. M0653T). Cas12a was diluted to its final concentration in 10X NEBuffer r2.1. crRNAs, RPA primers, synthetic target DNA and ssDNA-FQ were synthesised by Integrated DNA Technologies. All corresponding sequences can be found in Supplementary Table 2. Human urine was purchased from LeeBiosolutions (cat. No. 991-03-P-1). PrimeStore MTM from Longhorn Vaccines & Diagnostics and DNeasy purification kit from Qiagen (Cat. No. 69504) were used for nucleic acid extraction. The Axxin ISO T-16 portable fluorescence reader was used to take all real-time fluorescence measurements. Freeze-drying was performed using the FreeZone 2.5 L −50 °C benchtop freeze-drier by Labconco.

### Design of crRNA

crRNAs were designed using the CRISPOR online tool^53^. Briefly, the *S. haematobium* Dra1 repeat region sequence was submitted with parameters ‘TTT (A/C/G) Cas12a (Cpf1)—recommended, 23 bp guides’ for the Protospacer Adjacent Motif (PAM). The three crRNAs with the highest predicted efficiency and the lowest off-target score were chosen (Supplementary Table 2).

The crRNA sequences were designed to target *S. haematobium* but with an understanding that it will cross react with other *S. haematobium* group species which also contain the Dra1 repeat^47^.

### Preparation of Dra1 synthetic DNA, *Schistosoma* spp. genomic DNA and whole eggs

A dilution series of the synthetic double-stranded DNA (Supplementary Table 2) was prepared by diluting Dra1 synthetic DNA in nuclease-free water to 10^6^ copies/µL which was used as a positive control for CATSH optimisation. gDNA from *S. haematobium, S. mansoni*, *S. bovis* and *S. curassoni* adult worms, originating from the Schistosomiasis Collection a the Natural History Museum (SCAN)^54^, was provided as described in Rostron et al*.*^23^. Frozen *S. haematobium* eggs, provided by the Schistosomiasis Resource Centre (Biomedical Research Institute, San Antonio, TX, USA) were individually collected as described in Archer et al*.*^24^.

### CATSH assay optimisation

In a 0.2 mL PCR tube, the CATSH reaction was prepared by mixing three components we refer to as Component A (RPA reagents), Component B (ssDNA-FQ reporters) and Component C (CRISPR reagents).

Component A was prepared by rehydrating a pellet of TwistAmp Basic RPA with 29.5 µL of Rehydration Buffer, then adding 2.1 µL (at 10 µM) of both forward and reverse primers (Supplementary Table 2), and 13.8 µL of nuclease-free water. RPA inputs of 1× (7 µL of Component A) or 2× (14 µL of Component A) were tested.

Component B was optimised by using 2 µL of ssDNA-FQ at stock concentration 20, 50, 100 and 500 nM.

Component C was made as a 50 µL solution with Cas12a and crRNA, containing Cas12a to crRNA ratios of 1:1, 1:2, 2:1 or 2:2 ratios, where one part equals to 50 nM of reagent. Component C was made fresh and left at RT for 10 min prior to use allowing the crRNA-Cas12a complex to form.

One µL of MgOAc at 280 nM was pipetted into the lid of the tube. For positive control reactions, 1 µL of Dra1 synthetic DNA at 10^6^ copies/µL was used.

Tubes were briefly centrifuged to mix in the MgOAc which initiates the RPA reaction. The reactions were immediately incubated for 40 min at 37 °C and real-time fluorescence was measured using the portable reader.

### Final optimised CATSH assay

The optimised CATSH assay was prepared in a 0.2 mL PCR tube by mixing 14 µL of Component A, 2 µL of Component B at 100 nM ssDNA-FQ reporter, 2 µL of Component C with 1:2 Cas12a to crRNA1 ratio (50 nM Cas12a, 100 nM crRNA1) and 1 µL of MgOAc at 280 nM which was pipetted into the lid of the tube. Finally, the sample (1 µL for gDNA/egg or 5 µL for simulated urine sample) was added to the reaction. The tube was briefly centrifuged and incubated at 37˚C for 40 min in the portable fluorescence reader.

### Fluorescence data editing

Background fluorescence was subtracted from all fluorescence readings to normalise the runs and mitigate artefacts sometimes occurring at the beginning of the reading (e.g., bubbles). To do so, the fluorescence measurement directly preceding *t* = 60 s was identified, and all following measurements were subtracted by this value. In addition, *t* = 60 s was set as zero.

### Analytical sensitivity

The optimised CATSH assay was tested with 1 µL of *S. haematobium* gDNA at concentrations 10^–3^, 10^–2^, 10^–1^, 10^0^, 10^1^ and 10^2^ pg per reaction. Five repeats were done for each concentration. A fluorescence cut-off was calculated from five NTC reactions. After background subtraction, the endpoint (at *t* = 40 min) fluorescence of each NTC reaction (NTC_end_) were used to calculate the cut-off:

$$cut - off = mean\left( {NTCend} \right) + 3 \times stdev\left( {NTCend} \right)$$

Reactions were classified as positive if the endpoint background subtracted fluorescence was above the calculated cut-off (cut-off = 30). For each positive reaction, the time at which the signal crossed the cut-off was defined as ‘time to cut-off’.

Finally, the analytical sensitivity of the CATSH assay was defined as the concentration of *S. haematobium* gDNA per reaction that can be detected ≥ 95% of the time (< 5% false negative rate), also known as the effective concentration at 95% (EC_95_). The fraction positive (the percentage of positive repeats) for each concentration tested was calculated and plotted. Finally, a Probit regression was fitted to the fraction positive to determine the EC_95_ with the associated 95% confidence interval.

#### Species specificity

CATSH was tested with gDNA of *S. haematobium*, *S. mansoni*, *S. bovis* and *S. curassoni* to test for species specificity, as described in Rosser et al*.*^25^. An input of 1 ng of gDNA was added to the optimised CATSH reactions.

#### Simulated urine samples

To develop and validate a rapid and simple sample preparation method compatible with CATSH, we used simulated urine samples. The simulated samples were prepared by spiking 100 µL of human urine with a controlled number of frozen *S. haematobium* eggs^24^.

#### Commercial DNA extraction for urine samples

Two commercial DNA extraction kits for clinical sample preparation (DNeasy Blood & Tissue kit by Qiagen and SwiftX DNA by Xpedite) were used on urine samples containing 0, 1, 5 or 20 eggs.

The DNeasy Blood & Tissue protocol was followed by mixing 100 µL of urine sample with 200 µL of ATL buffer and 20 µL Proteinase K. This mix was incubated at 56˚C for 1 h. Then, 200 µL of AL buffer and 200 µL of ethanol were added. The mixture was pipetted into a DNeasy column and spin for 1 min at 6000×*g*. The column was placed in a new collection tube and add 500 µL of AW1 were added. The column spin for 1 min at 6000×*g*, then it was placed in a new collection tube and 500 µL of AW2 were added. The column spin for 3 min at top speed. The column was placed in new tube and 25 µL of AE buffer were added and incubated for 1 min, before a 1 min spin at 6000×*g*. This step was repeated once so the final product contained 50 µL.

The SwiftX DNA kit was used by mixing a 100 µL of urine simulated sample with two volumes (200 µL) of Buffer EN. 15 µL of Suspension A was added to the sample and mixed by vortexing. The tube was incubated at RT for 3 min, then transferred to a magnetic stand for 1 min. The supernatant was removed and discarded. 100 µL of Buffer DL was added to the tube and the magnetic particles were resuspended by vortexing. The tube was incubated at 95˚C for 5 min, then transferred to the magnetic stand for 1 min. The supernatant containing the extracted DNA was removed and transferred to a new tube.

After sample processing, 5 µl of product was added to the CATSH reactions and the final CATSH assay ran as described above. Unextracted crude urine samples (5 µL) containing 0 or 1 egg were also tested directly into CATSH reactions.

#### Rapid CRISPR-compatible DNA extraction for urine samples

The CATSH-compatible sample preparation was developed by mixing 100 µL of simulated urine samples (prepared as described above) with 100 µL of PrimeStore MTM, followed by a 5-min or 1-h incubation at RT.

After incubation, the urine-MTM sample was purified and concentrated using the DNeasy purification kit (Qiagen). First, the total 200 µL urine-MTM solution was loaded onto the purification column and centrifuged for 1 min at 6000×*g*. Then, 500 µL of AW1 wash buffer was added and the column was centrifuged for 1 min at 6000×*g*. A second wash step was done by adding 500 µL of AW2 wash buffer to the column and centrifuged for 3 min at 20,000×*g*. Lastly, 30 µL of elution buffer was added to the column, let sit for 1 min, then centrifuged for 1 min at 6000×*g*. This step was repeated once to increase DNA yield. To test the purified samples, 5 µL was added to CATSH reactions.

#### Lyophilisation of CATSH reactions

The final CATSH assay was prepared as described above, without the addition of MgOAc. The tubes were opened, sealed with parafilm and small perforations were made using a needle. The tubes were snap-frozen in liquid nitrogen and lyophilised (freeze-drying) for 4 h (temperature −50˚C, vacuum set point 0.025 mbar). The lyophilised reactions were resuspended just before use, by adding 18 µL of nuclease-free water, 1 µL of sample (10 pg gDNA) and 1 µL of MgOAc. The lyophilised reactions were packaged into heat-sealed metallic pouches containing a silica gel pack (Supplementary Fig. 3) and kept in a −20˚C freezer for long term storage. An oven was used to test the heat stability of the lyophilised reactions at 30˚C.

#### Analysis and software

Data plots were done using GraphPad Prism 9. Matlab (R2022a) was used to compute the Probit regression. Biorender.com was used to draw illustrations.

### Ethics

Human urine was purchased from LeeBiosolutions and was obtained from anonymised donors in an ethically-sound method.

## Supplementary Information

Supplementary Information.

## Data Availability

The datasets generated during and/or analysed during the current study, and the computer code used are available from the corresponding author on reasonable request, in line with the requirements of UCL and the funder (EPSRC policy framework on research data).
