# Supplementary material for: CRISPR-assisted test for Schistosoma haematobium
Source: Sci Rep. 2023 Mar 27;13:4990. doi: 10.1038/s41598-023-31238-y (PMC10042105; doi:10.1038/s41598-023-31238-y)
Supplement: Supplementary file 1 — Supplementary Information. [file 41598_2023_31238_MOESM1_ESM.docx]

**Supplementary information**

**CRISPR-assisted test for *Schistosoma haematobium***

Dounia Cherkaoui^1,2^*, Silvia G. Mesquita^3,4,5^, Da Huang^1^, Elena B. Lugli^3,5^, Bonnie L. Webster^3,5^* & Rachel A. McKendry^1,2^*

^1^ London Centre for Nanotechnology, University College London, London WC1H 0AH, UK

^2^ Division of Medicine, University College London, London WC1E 6BT, UK

^3^ Wolfson Wellcome Biomedical Laboratories, Department of Science, Natural History Museum, Cromwell Road, London SW7 5BD, UK

^4^ René Rachou Institute, Oswaldo Cruz Foundation, Belo Horizonte, Minas Gerais, Brazil

^5^ London Centre for Neglected Tropical Disease Research (LCNTDR), London W21 PG, UK

* Joint corresponding authors:

dounia.cherkaoui.17@ucl.ac.uk, b.webster@nhm.ac.uk and r.a.mckendry@ucl.ac.uk

**Supplementary Table 1.** Cost of CATSH reagents

| **Cost of CATSH reagents** | | | |
| --- | --- | --- | --- |
| **Reagents** | **Cost (£)** | **# Reactions** | **Cost/Reaction (£)** |
| **In-house extraction** | | | |
| MTM PrimeStore (Longhorn) | 241.1 | 500 | 0.5 |
| Dneasy kit (Qiagen) | 266.7 | 250 | 1.1 |
|  |  |  |  |
| Sub-total |  |  | 1.5 |
| **CRISPR assay** | | | |
| crRNA (IDT DNA) | 75 | 400 | 0.2 |
| *L.b.* Cas12 (New England Biolabs) | 184 | 400 | 0.5 |
| RPA kit (TwistDx) | 240 | 326.4 | 0.7 |
| RPA primers (IDT DNA) | 53.4 | >1000 | <0.1 |
| FD probe (IDT DNA) | 149.3 | >1000 | <0.1 |
| PCR tube (Starlab) | 19.3 | 1000 | <0.1 |
|  |  |  |  |
| Sub-total |  |  | 1.5 |
| **Total** |  |  | **3.0** |

**Supplementary Table 2.** Sequences for CATSH optimisation

| Component | Sequence |
| --- | --- |
| Forward primer | 5’-ATCTCACCTATCAGACGAAACAAAGAAAAT-3’ |
| Reverse primer | 5’-GTCGTATCGTTGTGAAAATTGTTTCATATT-3’ |
| crRNA1 | 5’-TTTAAAATTGTTGGTGGAAGTGCCTGT-3’ |
| crRNA2 | 5’-TTTAAAATTGTTGGTGGAAGTGCCT-3’ |
| crRNA3 | 5’-TTTCGCAATATCTCCGGAATGGTTG-3’ |
| ssDNA-FQ | 56-FAM/5’-TTATT-3’/3IABkFQ |
| Synthetic DNA | 5’-CCTTGGTCACGTGATTTTCAGTTTGCCCCACCCTGATGCTGGCTGCCCCAC  CTCGACCGGCATAAGGTGGAGCGATCTCACCTATCAGACGAAACAAAGAAAATT  TTAAAATTGTTGGTGGAAGTGCCTGTTTCGCAATATCTCCGGAATGGTTGGTCG  TATCGTTGTGAAAATTGTTTCATATTATTGGTGAC-3’ |


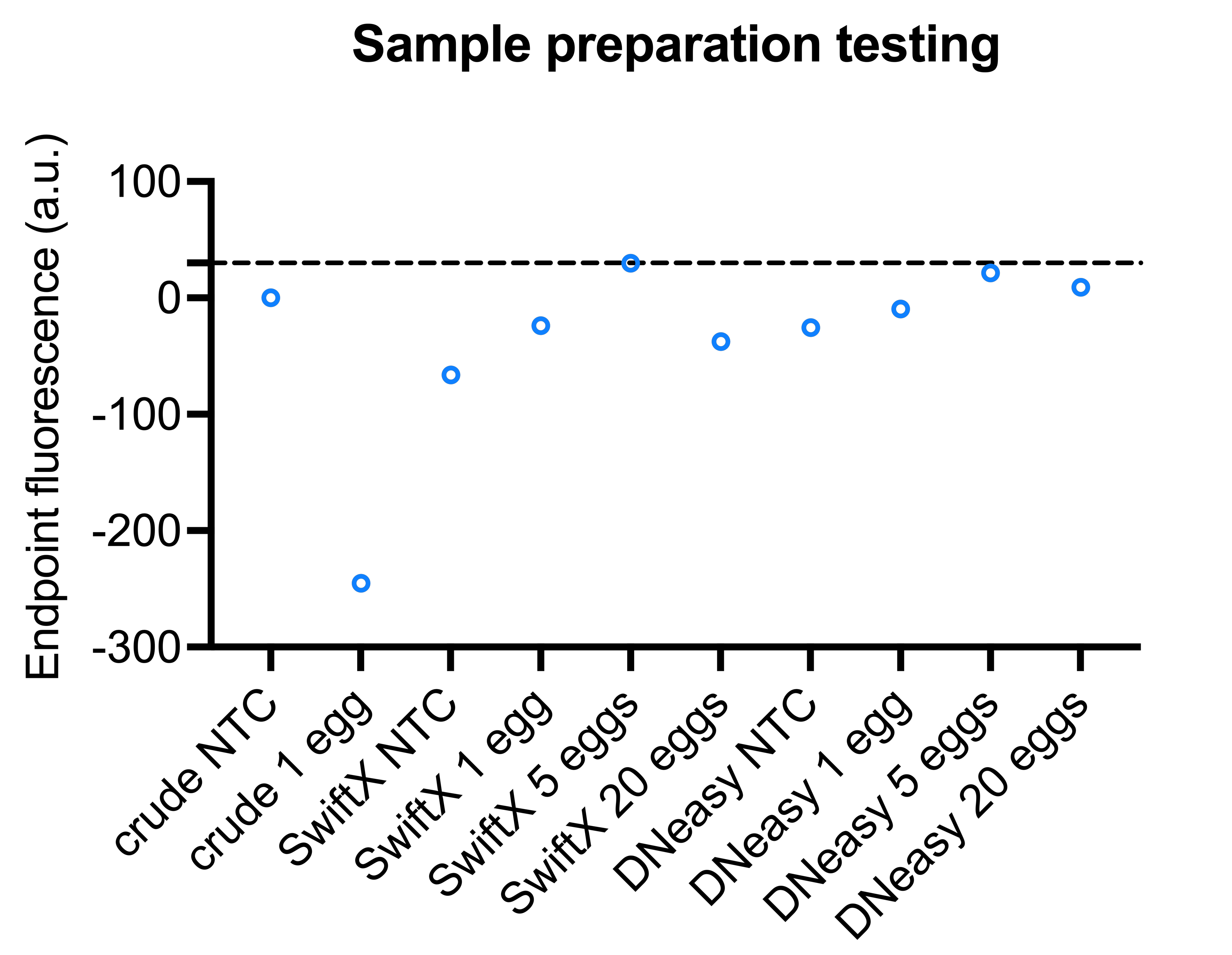


**Supplementary Figure 1.** Urine sample preparation testing

Crude simulated urine samples were tried directly in CATSH reactions. Two commercial kits (SwiftX DNA by Xpedite and DNeasy Blood & Tissue kit by Qiagen) were tried with different number of *S. haematobium* parasitic eggs. Each sample was tried once and each circle shows the endpoint fluorescence after background subtraction. This graph was plotted using GraphPad Prism version 9.0.


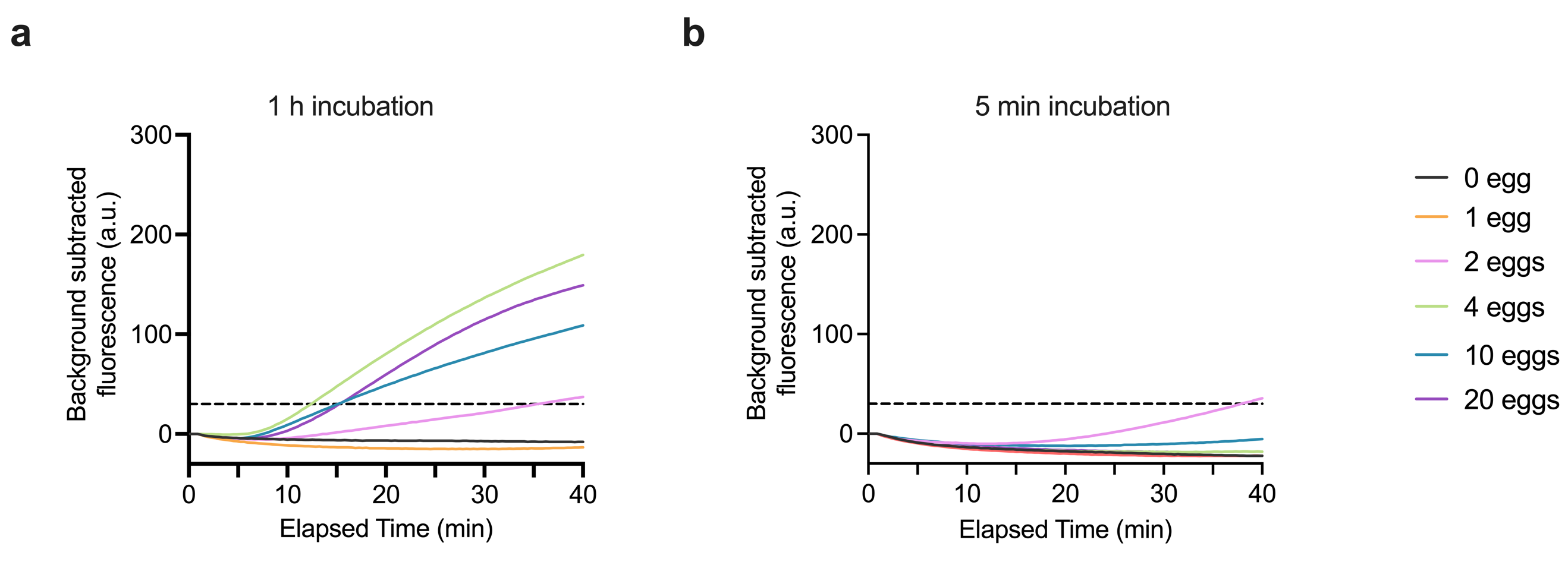


**Supplementary Figure 2.** In-house extraction of urine sample with long or short incubation with MTM

The in-house protocol developed for extraction of simulated urine sample (urine spiked with parasitic eggs) was tried with **a.** a long (1 hour) and **b.** short (5 minutes) incubation time with MTM. This graph was plotted using GraphPad Prism version 9.0.

**Supplementary Figure 3.** Thermostable packaging for freeze-dried CATSH reaction

Photograph of the packaging of the freeze-dried CATSH reactions. The freeze-dried reactions are stored with a silica gel pack to reduce humidity in a metallic pouch, which is heat sealed using a mini heat sealer.
